# Clinical Outcomes of the Implementation of IOP Monitoring, in and out of Office Time, to 1500 Patients—A Cohort Study

**DOI:** 10.3390/vision6040069

**Published:** 2022-11-21

**Authors:** Sevasti Tsironi, Diamantis Almaliotis, Panagiota Ntonti, Georgios Sidiropoulos, Evangelia Theodoridou, Efstratios Theofrastou, Sofia Karachrisafi, Eleni Psimenidou, Anastasia Sarafi, Victoria Kapourani, Frangeskos Loizou, Elie Fadel

**Affiliations:** Eye Clinic of General Hospital “George Papanikolaou”, 57010 Thessaloniki, Greece

**Keywords:** tonometric curve, IOP phasing, IOP monitoring, glaucoma, diurnal IOP, glaucoma suspects, IOP peaks, hypertension, compliance, adherence

## Abstract

Background: The aim of this study was to present the clinical outcomes of IOP monitoring in and out of office time and determine its value in our clinical practice. Material and methods: We reviewed the records of 1500 patients (glaucoma suspects or glaucoma patients), who were admitted for IOP monitoring during almost 12 years. All patients were hospitalized because their within-office-hours exams were considered inadequate and inconclusive for decision making. Results: A total of 744 patients (49.6% out of 1500) needed change of treatment. A total of 121 patients (8% out of 1500) were programmed for interventional therapy (laser or surgery). A total of 68 patients (4.5% out of 1500) were declassified as overdiagnosed and overtreated. In 250 patients (16.7% out of 1500), hidden adherence problem appeared. In 720 patients (48% out of 1500), peak IOP occurred during out-of-office hours. Conclusions: IOP phasing is a useful tool in clinical practice. In many cases with inconclusive diagnosis, as well as in patients with advanced or labile glaucomas, IOP monitoring data add complementary information, useful for decision making, and may contribute not only to diagnosis and successful IOP modulation, but also in personalized therapeutic strategy and individual patients’ motivation.

## 1. Introduction

Glaucoma is the leading cause of irreversible blindness worldwide, with 8.4 million sufferers resulting in total vision loss and 60 million with installed optic neuropathy. Those numbers are expected to rise in the years to come [1]. The etiology of glaucoma has not yet been determined. Its specific mechanism of onset has yet to be clarified. Yet, intraocular pressure (IOP) is considered the primum movens in glaucoma development. It is the greater risk factor and its reduction is the mainstay of treatment [2].

Sidler-Huguenin first reported the concept of diurnal IOP variation in 1898 and this was refined by Maslenikow in 1904. IOP varies considerably based on a number of factors, such as body position (supine vs. upright, fluid intake, endogenous cortisol, etc.). IOP variation on a circadian basis has been shown in healthy subjects [3] and in patients with a diagnosis of ocular hypertention (OHT) [4] or glaucoma [5]. Several papers have evaluated the 24 h IOP curves in patients untreated [6] or treated with different therapies [7,8,9] and in different settings (i.e., clinical or sleep laboratory) [3] and have all reported significant fluctuation.

Apparently, IOP is a dynamic rather than a stable biological parameter [10,11]. It is definitive for the disease course and the patient’s prognosis, since damage from high intraocular pressure is constant [12]. Therefore, a single measurement is insufficient to characterize the real IOP pattern and pathology [13].

Peak IOP and IOP fluctuations are known to be potential independent risk factors for the evolution of glaucoma [14,15]. Moreover, several studies showed that peak IOP may be recorded outside office hours [5,10,16,17,18,19].

In everyday practice, IOP monitoring is based on spot measurements during clinic visits in office hours [20,21], as there is currently no widely used clinical tool for continuous IOP monitoring. Those single, daily measurements are not necessarily the highest of the patient [10,15] and they also do not necessarily reflect the 24-hour fluctuation of intraocular pressure, which differs and varies widely in different patients, such as in cases of exfoliative glaucoma (XFG) and untreated POAG patients. For example, the higher measurements (peaks) in XFG occur at any hour and are significantly high [22], as well as the peaks of untreated POAG patients usually occurring between 6 a.m. and 10 a.m. [7].

The height of IOP and also mean IOP are regarded as risk factors for glaucoma progression. In the early Manifest Glaucoma Trial, each 1 mmHg increase in mean IOP over all follow-up visits was associated with a 12% higher risk of progression [23]. In the Advanced Glaucoma Intervention study, each 1 mmHg higher mean IOP level at the first 18 months of follow-up was associated with a 0.10 greater visual field defect score during the remainder of the follow-up. Furthermore, elevated mean IOP has also been found to be a significant risk factor for conversion from ocular hypertension to POAG [24,25]. Patients with adequate intraocular pressure control, specifically those with less than 18 mmHg in every measurement, had a significantly lower rate of developing visual field defects [26].

A broader intraocular pressure fluctuation is linked with a more rapid vision loss [27,28,29]. In addition, longer exposure to intraocular pressure fluctuation was significantly higher in patients suffering total vision loss due to glaucoma [30,31].

Yet, the role of peak IOP and fluctuation as independent risk factors in eyes with glaucoma is still not adequately defined [21,32,33,34,35,36]. Peak IOP may be clinically important in predicting long-term glaucomatous progression [14,33]. Previous studies suggest that IOP peaks occur outside office hours and go unnoticed [20,37,38]. Moreover, the detection of IOP peaks outside office hours has led, in some cases, to clinical management modifications that have included even filtration surgery [18,19].

In current practice, diagnosis and treatment decisions (even surgical) are usually based on several IOP measurements within office hours. Yet, long-term, continuous IOP monitoring represents the next giant leap forward in glaucoma management [37,39,40].

In our everyday clinical practice in the outpatient glaucoma unit, diagnosis is inconclusive for some patients (hypertensive, glaucoma suspects or glaucoma patients). Facing these clinical challenges, IOP monitoring can be of great value, as it allows a detailed and realistic IOP profile to be acquired, useful for diagnosis and treatment decisions, tailor-made management, and personalized patient guidance. Patients with serious risk factors as well as elevated IOP or suspicious optic nerve are often candidates for monitoring. The IOP pattern of these hypertensive or glaucoma suspects can be an aid for diagnosis, treatment choice, and even the timing of administration, accordingly. We also monitor glaucoma patients under therapy with disease progression, despite apparently “adequate IOP control” and being disproportionate to known office IOP measurements. For those, the question is if they are in need of treatment escalation or if their medication is sufficient but noncompliance is the problem. High-risk patients with advanced glaucoma, for whom a single daytime reading does not shine light on the IOP profile, are often prime candidates for IOP monitoring in an effort to identify out-of-office, unfavorable IOP characteristics. Finally, we monitor patients under therapy by other physicians and first examined in our outdoor clinic when considered overdiagnosed and overtreated.

This study aimed to describe our experience from the implementation of the IOP monitoring on the last 1500 hospitalized and recorded patients concerning their diagnosis and management. It is an attempt to identify the utility of IOP monitoring in real-life clinical practice.

## 2. Materials and Methods

A case–control study by medical record review was employed. The study protocol was approved by the study board of G.H.T. “G. Papanikolaou” and was conducted in accordance with the declaration of Helsinki. The study board of G.H.T. “G. Papanikolaou” did not require written informed consent obtained from the participants, as this was a retrospective study and all data were anonymous.

The research was conducted and completed at G.H.T. “G. Papanikolaou” Eye Clinic in Thessaloniki. The patients were retrieved from the eye clinic database. We included the last 1500 patients who had undergone diurnal IOP monitoring in the clinic until the conducting of the study. All of them were hospitalized between November 2007 and December 2019 at the Eye Clinic of General Hospital “George Papanikolaou” in Thessaloniki, Greece. Exclusion criteria included any patient with missing data values for IOP.

Intraocular pressure peaks (highest IOP recorded) were also monitored regarding time and incidence within and outside regular office hours [41]. Office hours were determined as 09:00–14:00 and 17:00–21:00. We deliberately set such an enlarged office-hour schedule, which includes not only the hospital office hours of outpatient units, but also the usual office hours of private clinics. This way we aimed to discover a percentage of IOP peaks, outside all possible, usual office hours of hospital and private eye clinics, in other words, outside the so-called “office diurnal curve”.

### 2.1. Participants

Patients were already monitored due to glaucoma or came for general control and were diagnosed as glaucoma suspects, either with OHT (IOP > 21 mmHg) or were, for some other reason, in danger of developing glaucoma. All of them were hospitalized for IOP monitoring according to a number of criteria followed in our clinic.

Inclusion criteria for IOP phasing consisted of glaucoma patients with intraocular pressure higher than target pressure despite being referred previously for sufficient therapy and, also, patients with equal or lower than target pressure but with disease progression. There were also included patients with advanced glaucoma with visual field defects on the visual field testing, as well as patients with normal tension glaucoma and labile secondary glaucomas (exfoliative, pigmentary, and chronic angle-closure glaucoma), for whom close monitoring and therapy reconsidering was judged appropriate. All patients were using their glaucoma medications when admitted and according to medical instructions prior to admittance. During hospitalization, the clinic staff was responsible for the medical therapy according to the pre-existing, individual schedule of every patient.

Glaucoma suspects, demonstrating findings consistent with increased risk for glaucoma development, were included. Such findings were: mostly OHT (IOP > 21 mmHg) but also suspicious optic disc (increased cup-to-disc ratio (>0.4), asymmetry (≥0.2) between optic discs, or both, as well as notching or narrowing of the neuroretinal rim) or glaucoma-positive family history (first-degree relatives with advanced glaucoma or even blind from glaucoma). Most suspects had more than one of the previous criteria. In Table 1, they are classified according to the most serious criterion individually.

We also included possibly overdiagnosed or overtreated patients who were first examined in our clinic and were already diagnosed as hypertensive or glaucoma patients and were given antiglaucoma therapy by other physicians. Their clinical examination in outpatient glaucoma clinic set the suspicion of overdiagnosis. Thus, we stopped their antiglaucoma therapy for one month and then hospitalized them for IOP monitoring.

Our aim was to create a more comprehensive, individualized pressure profile for each patient and use this information for decision making in diagnosis and management.

### 2.2. Procedure

For every patient a routine vision check with Snellen optotype was conducted, as well as slit lamp evaluation of the anterior part of the eye, gonioscopy with a 3-mirror Goldman lens, fundoscopy, central corneal thickness assessment, visual field analysis (Humphrey 30-2), and optic nerve OCT. We should highlight that almost all of the patients were already examined in the past, as mentioned above. Nevertheless, all exams were carried out anyway, with admission to hospital the same day of IOP phasing, regardless of time proximity of previous examinations. Moreover, 6 intraocular pressure measurements were conducted for both eyes. The IOP measurements were made by Goldmann applanation tonometer in a 24 h period and specific hours during the day (7.00, 10.00, 13.00, 16.00, 19.00, and 22.00), with the patient in the sitting position. The Goldmann tonometer was calibrated at the beginning of each day. One drop of 0.5% proparacaine eye drops was used for topical anesthesia and the tear film was stained with fluorescein strips. A third IOP measurement was performed if a difference higher than 2 mmHg was found between the first two measurements. The IOP value obtained was an average of the two closest IOP measurements.

## 3. Results

**Figure 1:**
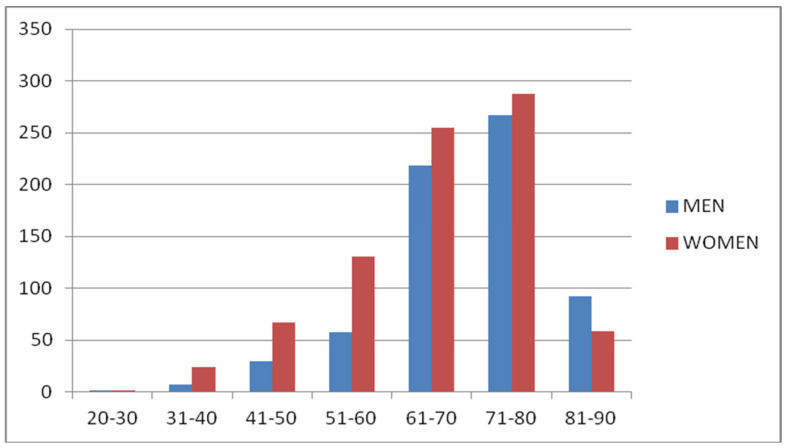
Age distribution of glaucoma patients based on gender.

In total, 1500 patients populated the study group, 674 males and 826 females. A total of 548 of them had primary open-angle glaucoma (POAG), 249 ocular hypertension (OHT), 16 chronic angle-closure glaucoma (ACG), 218 exfoliation glaucoma (XFG), 88 exfoliation syndrome (XFS), 48 normal pressure glaucoma (NTG), 246 increased or asymmetrical c/d ratio or both (C/D), 85 had serious glaucoma family history (GFH), and 2 had pigmentary dispersion syndrome (PDS). Specific demographics in Table 1. The age range was between 30 and 90, with the majority between the ages of 61–70 and 71–80 regardless of gender in Figure 1.

**Figure 2:**
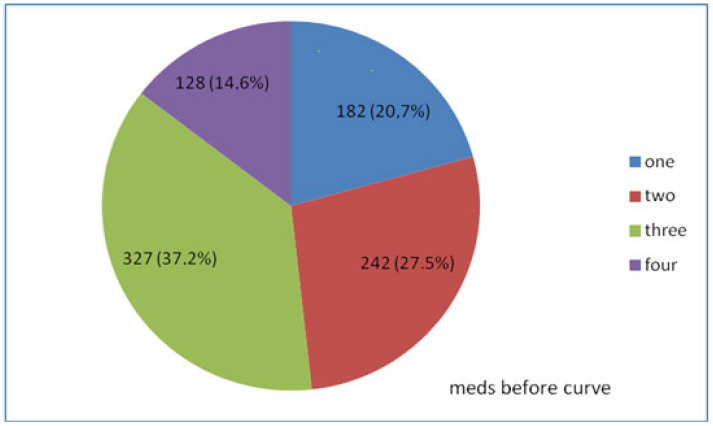
Treatment based on the number of medications taken before IOP monitoring of 879 patients.

Before entering hospital for IOP monitoring, 879 (58.6%) of the 1500 patients were under medical therapy with antiglaucoma meds, while the remaining 621 (41.4%) did not receive any antiglaucoma medical treatment. From the 879 patients under therapy 182 (20.7%) were treated with one antiglaucoma agent, while the remaining 697 (79.3%) with two, three, or four antiglaucoma agents, either single or in combination (Figure 2).

The main goal of the patient’s IOP monitoring was to gain more information concerning IOP characteristics in favor of our diagnostic and therapeutic strategy.

Taking into consideration the IOP monitoring results, 744 monitored patients (49.6% of all 1500 patients and 84.6% of 879 patients already under meds) needed change of treatment.

In 157 patients (10.4% of total 1500), commencement of treatment was decided. In other words, out of 621 patients who did not receive any therapy, 157 (25.3%) began antiglaucoma therapy after IOP monitoring.

Out of 879 patients who were taking meds prior to IOP phasing, several treatment changes were made based on IOP monitoring data. In 311 (20.7% of 1500, 35.4% of 879) of them, their drug was changed to another agent. In 87 (5.8% of 1500, 9.95% of 879), another drug was added to previous therapy. Moreover, 68 (4.5% of 1500, 7.7% of 879) patients were declassified as overdiagnosed and their antiglaucoma treatment was considered unnecessary and, therefore, stopped.

A total of 12 patients (1.2% of 1500, 1.4% of 879) were programmed for laser treatment and, for 109 (7.2% of 1500, 12.4% of 879), surgical treatment was suggested. So, for a total of 121 patients (8% of total 1500, 13.8% of 879 patients under antiglaucomatous therapy), medical therapy was not acceptable anymore and interventional therapy was needed. Due to information from IOP monitoring, interventional therapy was decided and suggested in almost 14 out of 100 patients under medical therapy.

**Figure 3:**
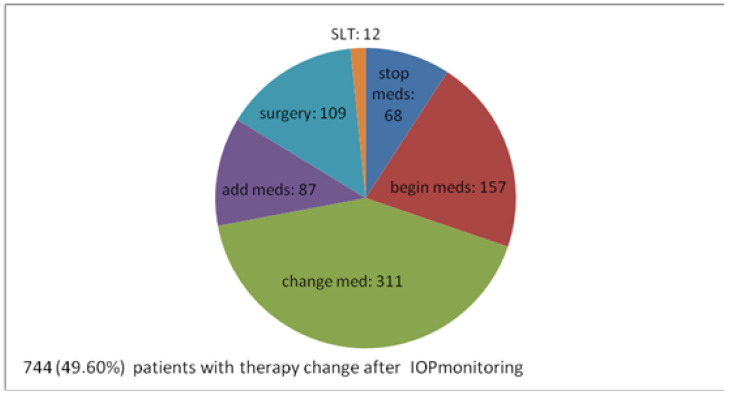
Alterations of the therapy after IOP monitoring—numerical distribution.

Overall, IOP monitoring helped physicians come to the clinical decision that 744 (49.6% of all 1500 patients and 84.6% of 879 patients already on medical antiglaucoma therapy) needed a change of treatment, meaning either beginning therapy or stopping therapy or having a different one, medical or surgical (Figure 3).

In total, 756 (50.4% of 1500) patients needed no changes regarding their therapeutic management. Of these, 464 (30.9% of all 1500, 61.4% of 756 patients needing no therapeutical change) remained in observation in order to attend to any glaucoma manifestations. A total of 292 patients already on antiglaucoma medication (19.5% of 1500, 38.6% of 756) remained with the same medication, which was considered satisfactory, according to IOP measurements.

**Figure 4:**
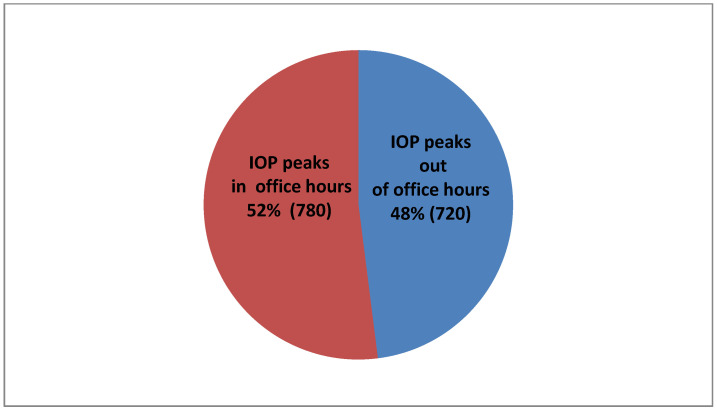
IOP peak percentages in conjunction with the office hours.

As far as distribution of IOP peaks is concerned, in 780 of our patients (52% of 1500), their peak IOP was measured within office hours and 720 (48% of 1500) occurred out of office hours (Figure 4).

**Figure 5:**
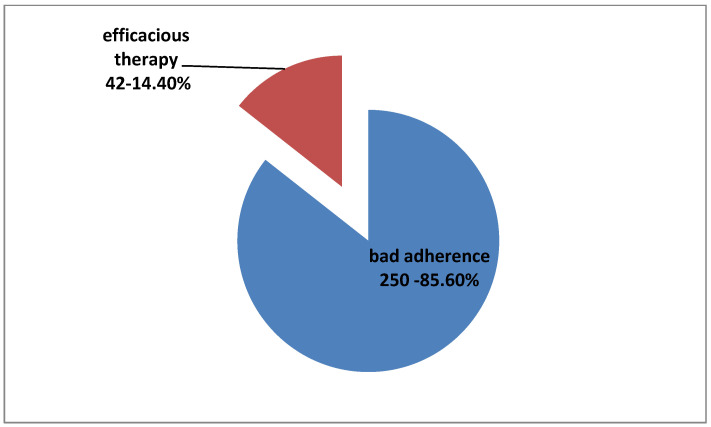
Percentage of bad adherence among 250 patients with no change in therapy disclosed due to IOP phasing procedure.

A total of 250 patients (16.7% of total 1500, 28.4% of 879 patients on medication before IOP monitoring,) were in no need for antiglaucoma medical treatment change but, during their hospitalization, an adherence problem was revealed. IOP monitoring showed a satisfying control of their IOP into hospital. Their previous IOP measurements in outpatient glaucoma unit examination were either higher than target pressure, satisfying according to target pressure but with worsening of clinical examination (vision and optic nerve), and/or OCT and/or VF examinations. That inconsistent, mismatched, and, finally, inconclusive diagnosis had led them to hospitalization and IOP monitoring in order to solve the diagnostic and therapeutic problem. At this point, we should clarify that, in general, all of our patients are informed about the nature of glaucoma disease and receive detailed and practical advice about the method and the schedule of their antiglaucoma drops instillation. Furthermore, all patients examined in outpatient glaucoma unit are questioned about their adherence and possible practical problems with medications, and they receive all possible aid, individually, when problems occur and bad adherence is noted. All patients on medical therapy included in our study had claimed good adherence when questioned during their regular outpatient examinations before IOP curve programming (Figure 5).

## 4. Discussion

The purpose of this study is the description, quantification, and evaluation of our experience in the clinical management of glaucoma patients or suspects by use of IOP monitoring. Specifically, our objective was to discover whether it was beneficial and helpful when regular examination in the outpatient unit was not adequate for decision making in several cases, such as: patients with probable ocular hypertension, glaucoma suspects (optic nerve characteristics, family history, XFS, and PDS), patients with progressive glaucoma despite normal single office IOP measurements, high-risk patients with advanced glaucoma (where detailed IOP monitoring is definitely needed), patients with normal tension glaucoma and labile secondary glaucomas (exfoliative, pigmentary, and chronic angle-closure glaucoma), for whom close monitoring and therapy reconsideration was judged appropriate, patients with suspected hidden bad adherence, and, finally, patients suspected as overdiagnosed and/or overtreated.

According to the literature, IOP monitoring holds high significance within the treatment of glaucoma [33]. It can offer assistance in obtaining a true picture of IOP levels for a glaucoma suspect but, also, in clarifying the progression of glaucoma patients with apparently “adequate IOP control”. If significant peaks are noted, IOP fluctuations may be responsible for the pathological alterations associated with glaucoma [14,32]. Yet, several investigators claim that there is low-quality evidence regarding whether the use of a diurnal tension curve is beneficial.

In this study, IOP monitoring helped us modify the first uncertain, therapeutic approach in many cases with inconclusive diagnosis. This was obvious both in cases of making a new diagnosis and commencement of treatment (157 patients, 10.4% of total 1500), as well as in 68 declassified cases that actually did not require therapy but were already being treated (68 patients, 4.5% of 1500, 7.7% out of 879 patients who were taking medication before IOP monitoring). By revealing hidden peaks of IOP in glaucoma suspects, IOP monitoring helped in the identification of true IOP characteristics and better delineation of the risk for glaucoma progression. A more comprehensive, individualized profile for each glaucoma suspect helped us detect the thin line that separates the indication for observation from indication for treatment. Identifying patients’ individual IOP pattern not only facilitated the diagnosis, but also directed choice of treatment. In overdiagnosed and overtreated patients, IOP monitoring without treatment confirmed nonpathological IOP profile and ensured treatment discontinuation and patients’ declassification, on a more robust database.

IOP monitoring and evaluation of IOP characteristics showed us that 744 patients (49.6% of all 1500 patients and 84.6% of 879 patients under medical antiglaucoma therapy) needed a change of therapy, either beginning therapy or stopping therapy or having an interventional therapy, medical or surgical. IOP monitoring outside of normal office hours has already been reported to lead to treatment change for many patients (30% to 80%), suggesting important clinical utility of IOP monitoring [18,19].

As far as IOP peaks, in 720 patients (48% of 1500), they occurred outside office hours and added useful information in benefit of our diagnosis and treatment rationale. Our results are in accordance with several studies in the literature. In a prospective study [7] of 80 patients with newly diagnosed untreated exfoliative glaucoma and primary open-angle glaucoma, the investigators observed elevated peak IOP values outside office hours in up to 45% of exfoliative glaucoma and 22.5% of primary open-angle glaucoma patients. Several studies also highlighted that the highest IOP measurement was mostly (42% to 66%) obtained outside office hours [19,36,42,43]. In a study comparing advanced POAG patients successfully treated with trabeculectomy and patients considered to be well controlled on maximal medical therapy, most IOP peaks (10 of 11) occurred outside usual office hours [36].

Daytime peak IOP may be clinically important in predicting long-term glaucomatous progression [14,15]. Therefore, when IOP is not measured outside office hours, these peaks cannot be documented, creating negative effects in the long-term treatment of the disease. At this point, we should comment that the definition of typical office hours mildly variates among studies. However, monitoring IOP outside any usual office hour schedule selected in the literature showed that we detect a remarkable percentage of IOP peaks, which would be missed by any office hour monitoring only, either single or repeatedly.

Another important finding was the role of IOP monitoring in the revelation of hidden bad adherence, thus avoiding overtreatment and educating and helping appropriately patients with poor compliance. In total, 250 patients already receiving treatment (16.7% of total 1500, 28.4% of 879 patients on medication before monitoring, 33% of 756 patients who did not need therapeutic strategy change, and 85.6% of 292 patients who should remain on the same medical treatment, as it was efficacious considering monitoring) did not instill medications correctly and, consequently, maximum efficacy of the medication could not be reached. At this point, it is important to highlight that almost all of our patients were already informed about the disease’s gravity and the importance of antiglaucoma therapy and claimed good adherence when routinely examined and questioned in our outpatient glaucoma unit. Their bad adherence was disclosed due to IOP monitoring procedure because, during the hospitalization, medications were administered by our nursing staff. In all these cases, after monitoring, patients were thoroughly reinformed on the seriousness of glaucoma disease and diligently educated for the correct use of their medication. Their individual characteristics and daily program were taken into account and their medical therapy schedule was simplified, if possible. Reminders were suggested in order to facilitate the therapeutic program, as well as help from available people from the patients’ environment. They were also informed on possible alternative therapies, such as lasers or surgery, if their characteristics were proper. Personalized approaches were implemented and we did not only add unnecessary medications but, instead, we individually educated them in order to better understand their disease and apply their treatment effectively. Moreover, individually, we tried to find the best therapeutic alternatives and to personally motivate and involve them, conserving their glaucoma therapy.

Due to IOP monitoring, clinical management actively changed in 994 patients (66.3% of total 1500), not only in the 744 for whom we changed therapy (stopping medication, beginning medication, changing medication, adding medication, or programming laser or surgery), but also in 250 patients for whom hidden, bad adherence was revealed and more active advisory support and endorsement was offered. The fact that IOP monitoring findings led us in differentiating our diagnosis and treatment to almost 70% of patients examined seems to clearly justify its implementation in our clinical practice. For the majority of our patients, IOP profile was the critical particle of the diagnostic puzzle in order to form an individualized therapeutic approach [44].

IOP is a dynamic biological parameter [10,11,45], affected by circadian rhythm, with unpredicted long-term or short-term variants [10,11]. Interestingly, short- and long-term IOP fluctuation are influenced by various factors, including blood pressure, heart rate, breathing, accommodation, eyelid blink, pupillary size, eye movement, central venous pressure, ingestion of water and osmolarity, sleep, postural changes, physical activity, and hormonal factors. Moreover, temperature-related and seasonal variations in IOP have also been reported [46]. However, it is not fully understood how IOP changes and how it works.

Nevertheless, to date, IOP remains critical and usually the main modifiable risk factor for the development and progression of glaucoma. Crucially, IOP is not fixed but varies considerably during the day and between one visit and another. IOP characteristics are considered important for disease evolution [11,27,33,35]. Consequently, a single IOP measurement during so-called office hours is insufficient to characterize the real IOP pathology of a patient with glaucoma [10,33]. Obviously, in clinical practice, we try to make all possible efforts to collect the best available information on IOP characteristics in order to ensure the best diagnosis and treatment for patients. Towards this direction, IOP monitoring and subsequent IOP data collection may be helpful. Nevertheless, hospitalization is rather costly for health systems and burdensome for patients and clinicians and, therefore, it cannot be suggested as a regular and repeatable examination for all glaucoma suspects or patients. Several IOP measurements at different day hours, even on different days, could provide useful information for IOP height or fluctuation.

IOP monitoring has been claimed to be an essential tool concerning glaucoma study for over 50 years. However, its necessity is debatable. Currently available literature does not provide answers to several key issues concerning the role of IOP characteristics in glaucoma and our ability to record and interpret these in a clinically meaningful way [10,33]. It is difficult to compare the outcomes of diurnal variation between various studies. Differences in clinical settings, including medication, position, time points, and the use of different tonometers, may contribute altogether to the differences. Nevertheless, most investigators tend to agree with the opinion that apparently disparate results of several studies are not conflicted but, rather, can be viewed as complementary [32,33,45]. Prospective studies investigating individualized significance of intraocular pressure curves for therapeutic decisions could verify their utility [47]. Now, 24 h continuous IOP monitoring can be performed using a contact lens sensor. Studies have shown the good safety and reproducibility of this device. Better knowledge about IOP patterns probably could help to better diagnosis and manage glaucoma, provide individualized disease management, improved adherence, and generally better treatment outcomes [11,13,33].

Despite our large cohort, the retrospective design of our study is an obvious limitation. We also realize that, although all the IOP measurements were obtained by a trained resident, there could have been some inter-observer bias in our results.

## 5. Conclusions

This analysis provides a “real-life” account of IOP in a large number of glaucoma suspects and patients. Our results demonstrate the potential for significant additional information being obtained from IOP monitoring in clinical practice. In glaucoma suspects, the true characteristics of the IOP curve become evident before the pressure curve is altered by the commencement of treatment. In treated patients, the real efficacy of therapy and the quality of IOP control becomes evident.

As a conclusion, it can be highlighted that IOP monitoring could be beneficial for diagnostic thinking and for therapeutic decision. Probably, it could facilitate not only IOP modulation, but also personalized therapeutic strategy and patients’ motivation.

Considering the difficulties of hospitalization and resources, IOP monitoring could realistically be used in clinical practice as a diagnostic tool in glaucoma suspects or glaucoma patients, for whom diagnosis is uncertain and inconclusive based on single office- hour IOP measurements.

However, prospective longitudinal studies are necessary to evaluate the clinical usefulness and value of diurnal curve, while expecting future technology tο discover and ensure safe, affordable, noninvasive, continuous, automatic, and true IOP measurement.

## Figures and Tables

**Table 1:** Incidences of different reasons for conducting diurnal curve in relation to gender.

Condition	OHT	POAG	ACG	XFG	XFS	NTG	C/D	GFH	PDS	Total
**men**	59	323	4	117	39	24	95	11	2	674
**women**	190	225	12	101	49	24	151	74	0	826
**total**	249	548	16	218	88	48	246	85	2	1500

OHT: ocular hypertension, POAG: primary open-angle glaucoma, ACG: angle-closure glaucoma, XFG: exfoliative glaucoma, XFS: exfoliative syndrome, NTG: normal tension glaucoma, C/D: suspicious cup-to-disc ratio, GFH: glaucoma family history, PDS: pigment dispersion syndrome.

## Data Availability

The data used to support the findings of this study are available from the corresponding author upon request.
